# Remarks on the Medicinal Properties of the Croton Oil

**Published:** 1825-08

**Authors:** Ed. Tegart

**Affiliations:** Inspector of Hospitals, &c. &c.


### Art. IV.—*Remarks on the Medicinal Properties of the Croton Oil.* By Ed. Tegart, Esq. Inspector of Hospitals, &c. &c.

*London; 22d May,* 1825.

Sir,

Regular and liberal-minded medical men have ever considered it a duty they owe to their professional brethren, as well as to society in general, to communicate whatever (whether it may be the result of scientific research and discovery, or useful practical experience,) they may conceive beneficial to mankind, in preserving health, in alleviating pain, or in curing disease. Acting upon this principle, I feel myself called on to state, that I have witnessed so much benefit from the use of a medicine imported into this country by you a few years ago,—I mean croton-oil,—that I cannot, in justice, withhold from you the high opinion I entertain of its great efficacy in many diseases.

Early in 1821, which was soon after the croton-oil became known in England, I was appointed to the superintendence of the medical department in the West Indies; and, as I had seen some, and heard of many extraordinary cases of its powerful effects as a purgative medicine, I was forcibly impressed that it would prove an important remedy in the diseases of that country. I therefore took out with me a supply, in order to give it a fair trial. I take credit to myself for having been the first who introduced it into that part of the world, the diseases of which it appeared to me so eminently calculated to oppose. I

sent a small quantity to all the colonies and islands, with a circular letter addressed to the principal medical officers; a copy of which I shall subjoin to this statement. I will also add an extract of a letter from me to the director-general of the army medical department, on receiving the required communications from those officers.

The Reports from the different stations fully confirmed the favourable opinion I had formed of this medicine. These documents were sent by me to the Board at home; and, should you wish to see them, or to obtain extracts from them, you will find every facility, the director-general and principal inspector being at all times desirous of imparting to the profession, and to the public at large, any and every part of the important information which they are constantly receiving from their officers stationed in every part of the world occupied by British troops.

Besides the official Reports from the army medical officers, numerous cases were transmitted by them, as well as civil practitioners, which I now regret were left at Barbadoes, because they might have been useful, as furnishing undoubted testimonials of the variety of diseases in which the croton-oil was employed, and the success which attended its exhibition It not only proved an active remedy in acute affections requiring depletion, but a stimulant one also in chronic visceral complaints: even when water had been deposited in the abdomen and chest, it acted as a powerful hydrogogue.

Dr. Lefort, physician to the King of France, and chief of the medical staff in the French West-India colonies, having heard of the success of this medicine in our islands, requested from me a small quantity. He was so much satisfied with its effects, that he immediately sent to London for a large supply: he has, no doubt, reported its success to his own government. This gentleman has lately published an excellent work on Yellow Fever, and, as he is an able and scientific man, he will soon discover that croton-oil is not to be confined solely as a purgative, and he will administer it, as we have done successfully, not only in our military hospitals, but by the private practitioners throughout our colonies, in all cases requiring depletion, either by the alimentary canal, kidneys, or skin.

When I first saw the croton-oil prescribed in England, it was given with the sole view of emptying the bowels; but experience in the West Indies soon convinced me that it was an excellent febrifuge medicine, after the first object had been attained. Dissolving it in spirits of wine, and then diluting it in any palatable vehicle, so as to give it in the proportion of about half a drop for a dose, every three or four hours, it keeps the bowels open, increases the urinary discharge, and relaxes the skin: these are important objects to gain at the commencement of all febrile affections.

Pyrexial attacks in tropical climates, but especially amongst soldiers and sailors, who, from intemperance and irregularity, are exposed and predisposed to such, are almost always attended with torpid bowels, constricted skin, and great determination of blood to the head. In such cases, the indications, of course, are to remove those symptoms; and I have seldom or never seen two or three drops of croton-oil fail in effecting that end. The lancet and warm baths are, however, generally employed at the same time in such cases. I have never known this oil disappoint my views when given as a purgative, and administered in proper time; but I have known some cases, and have heard of many, in which it has failed to operate. I am inclined to think those failures happened from too long delay, and that gangrene had taken place. Certainly I have known it act powerfully in many cases where calomel, scammony, gamboge, and other drastic purges, have been employed in vain.

With the exception of apoplexy and coup de soleil, I am not aware of any other disorder in which the croton-oil is employed with more evident effect, than in yellow fever. In this disease there is generally incessant vomiting, with obstinate constipation of the intestinal canal. To remove this obstruction, is a most important measure with the medical attendant: but how is he to accomplish this, as no medicine hitherto discovered could be retained on the irritable stomach? Two or three drops of this oil, applied to the tongue, will soon find its way to and through the stomach, thereby relieving that organ, by giving another direction to its morbid secretions. To relieve the sufferings, or to lessen the ravages of this formidable disease, would be a desideratum in our art. No remedy, that I have yet heard of, has done, or is likely to do, so much good as the one in question. In short, I think so highly of it, and consider it so valuable a medicine, that I have recommended it to be (and I hope it will be,) a permanent article in all military medicine chests.

In concluding this letter, I would beg to suggest to you, that every possible precaution may be taken by you to prevent the adulteration of the croton-oil, as I am informed that, in many instances, ten or fifteen drops have been given to produce the desired effect. You will, I am sure, best consult your own advantage, as well as public benefit, by preventing such wicked and dishonest practices on the part of the retailers of this medicine. I would further recommend that glass stoppers may be used instead of corks for the phials, as it is often found difficult to extract these corks, which are frequently decayed, probably from the action of the oil.

I am, Sir, your very obedient, humble servant,

EDW. TEGART,

*Inspector of Army Hospitals.*

To ARTHUR SHORT, Esq.
No. 11, Ratcliff Highway, London.

### No. I.

*Copy of a Circular Letter from* Inspector of Hospitals TEGART, *to the principal Medical Officers of Stations in the Windward and Leeward Islands.*

*Head-quarters, Barbadoes; June 21st,* 1821.

Sir,—Herewith I send you a small phial of the oil of the croton nut, which has been lately imported into England from the East Indies. In that country it was found eminently useful as a purgative medicine, exciting the secretions, relaxing the skin, and acting on the whole system as a most efficient febrifuge.

It has been used in the public hospitals and institutions of London, with decided success. I myself have exhibited it, and seen it given by others, with an astonishing good effect. The facility of conveying it into the stomach (one drop being a dose), and the quickness of its action, will render it a most important remedy in our materia medica. In apoplexy, I have known a patient, insensible and incapable of swallowing, purged and relieved in one hour, by merely putting one drop of this oil on the tongue.

From what I saw of this medicine, I augured most favourably of its powers in the ardent remittent and yellow fever of the West Indies. I was therefore induced to bring with me a small quantity, for the purpose of trying its efficacy. Should the result prove favourable, I will make it known to the Board, and request that a supply may be sent out for the use of the colonies.

The dose of this oil is from one to three drops; I have generally given two. In severe and sudden cases, such as apoplexy and coup de soleil, two drops on the tongue will be found sufficient. When there is irritability of stomach, it will be proper to give it in two ounces of peppermint-water, (rubbed down first with a little mucilage;) for it sometimes creates nausea and sickness. When given alone, it excites great heat in the mouth, which lasts for a considerable time.

How this oil acts, will afford ample subject for physiological discussion. From the sudden stimulating effect it produces, when applied to the epidermis; from the warmth it communicates to the œsophagus and stomach, and from the increase of the peristaltic motion, we may suppose it acts directly on the sensorium. I have witnessed a case of tic doloureux cured by it, when given with the intention only of removing a torpor of the bowels, which usually attends that disease.

In making this statement, it is far from my wish to create a prejudice in favour of this medicine. I am anxious to have it fairly tried, and reported upon. I am to beg you will make a selection of a few cases, watch attentively the effects, and give me your opinion. I trust it may be a long time before you will have an opportunity of giving the croton-oil in yellow fever; but other fevers we may expect to witness daily in this climate, which, if benefited by this means, may give fair grounds to hope that it may be advantageously employed when that formidable disease again appears amongst us.

I have to request you will, with as little delay as possible, make this experiment at ——; and communicate to me the result, by detailing a few cases, together with the form in which you have ordered the medicine. It will be but fair to aid its operation with the lancet, which, I believe, is now generally practised in all recent attacks of fever. It will be well, also, not to confine the experiment to acute diseases; because I think the remedy will be found very useful in chronic cases, particularly where visceral obstruction exists. I shall not fail in apprising you of the collected opinions on this subject, after the reports arrive at head-quarters.

I have the honour to be, Sir, your most obedient, humble servant,

(Signed) EDW. TEGART,

*Inspector of Hospitals.*

### No. II.

*Extract of a Letter from* Inspector of Hospitals TEGART, *to* Sir JAMES M'GRIGOR, Director-General Army Medical Department.

*Barbadoes; 30th November,* 1821.

A short time before I left London, a purgative oil, of a very extraordinary character, had been imported from the East Indies: the qualities ascribed to it were such, that I thought it my duty to give it a trial in this country. I purchased about thirty bottles for that purpose; and, soon after my arrival, I sent one or two to each of the islands and colonies in the command, in order that its effects might be ascertained, and reported upon. Herewith I have the honour of transmitting a copy of the circular letter written to the principal medical officers on the occasion.

As only very few have reported on the croton-oil, I will withhold them until the others arrive, and send them all at the same time. I will now merely send for your perusal a few extracts from letters on the subject, by which you will see that this medicine is highly estimated and extolled by some, although not noticed by others. For my part, my mind had been decided on its powers before I left England; and I have reason to think I shall not be disappointed in my expectations of it in this country. It is a remedy that, I think, will make its way, in spite of prejudice and opposition; and it has many opponents,—some from, perhaps, commendable caution, in not using a medicine until time and practice have ascertained its effects; and others from prejudice in favour of their own particular remedies, which long practice has convinced them that no others can equal or surpass.

The croton-oil will, I think, be found a most powerful remedy in many diseases, both in high and low latitudes: between the tropics, I think, it will be particularly so, from the rapidity of its action on diseases where delay is death. The facility of administering it, and the certainty of its action, are strong in its favour. It is now much known in this island, and I have had frequent applications from the inhabitants for a little, but my means of supplying are now very limited, as I have only a few bottles left. I did not intend making a requisition until I could accompany it with the reports from the out stations; but, as they are not all arrived, and as my stock is so small, I am under the necessity of requesting that you will be pleased to direct a supply of one hundred bottles, to be sent out by the packet, should the yearly supply of medicines have left England before this arrives; but, if they are not yet embarked, the croton-oil may be forwarded at the same time. I enclose the address of the person in London from whom I purchased what I brought to this country, and who is the only person that sells it.

### No. III.

*Extract from* Inspector of Hospital TEGART's *Annual Report for* 1823, *addressed to the Army Medical Board.*

*Barbadoes; 28th February*, 1824.

There are very few of the annual or occasional Reports from the colonies, that do not record fresh instances of the powerful effects from this medicine (croton oil). It is gaining ground in reputation every day, and will ultimately overcome (as I predicted last year,) the prejudices which were entertained against it. The smallness of the dose, and its active operation, occasioned fears in the minds of those who had not seen it given: these alarms have now ceased, and it is considered perfectly safe. The heat and irritation in the mouth and fauces are against its exhibition; but what man of sense would hesitate, from so trifling an inconvenience, to administer it in an important and dangerous disease.

I always feel gratified in reflecting that I introduced this remedy into this country, where there is such a field for displaying its powers. The best and most flattering account I have yet seen of its efficacy, is from the chief of the medical staff of the French islands, Dr. Lefort, who has requested me to order him a supply to be sent from London to Martinique. He recommends its exhibition in form of pills, to prevent the burning sensation when given in a liquid state. I began its use by dropping it on the tongue, and, having witnessed in that way its constant and unvaried success, I am unwilling to alter the mode of exhibition. I should apprehend it might not have the active and immediate effect, if it did not come at once into contact with the epidermis and lining membranes of the stomach.

